# Three-Dimensional Monolithically Self-Grown Metal Oxide Highly Dense Nanonetworks as Free-Standing High-Capacity Anodes for Lithium-Ion Batteries

**DOI:** 10.1021/acsami.2c05902

**Published:** 2022-06-14

**Authors:** Adam Cohen, Nimrod Harpak, Yonatan Juhl, Pini Shekhter, Sergei Remennik, Fernando Patolsky

**Affiliations:** † Department of Materials Science and Engineering, the Iby and Aladar Fleischman Faculty of Engineering, Tel Aviv University, Tel Aviv 69978, Israel; ‡ School of Chemistry, Faculty of Exact Sciences, Tel Aviv University, Tel Aviv 69978, Israel; § Wolfson Applied Materials Research Centre, Tel Aviv University, Tel Aviv 69978, Israel; ∥ The Center for Nanoscience & Nanotechnology, Edmond J. Safra Campus, The Hebrew University of Jerusalem, Jerusalem 91904, Israel

**Keywords:** manganese oxide, stainless steel, anode, lithium ion, transition metal oxides, energy storage

## Abstract

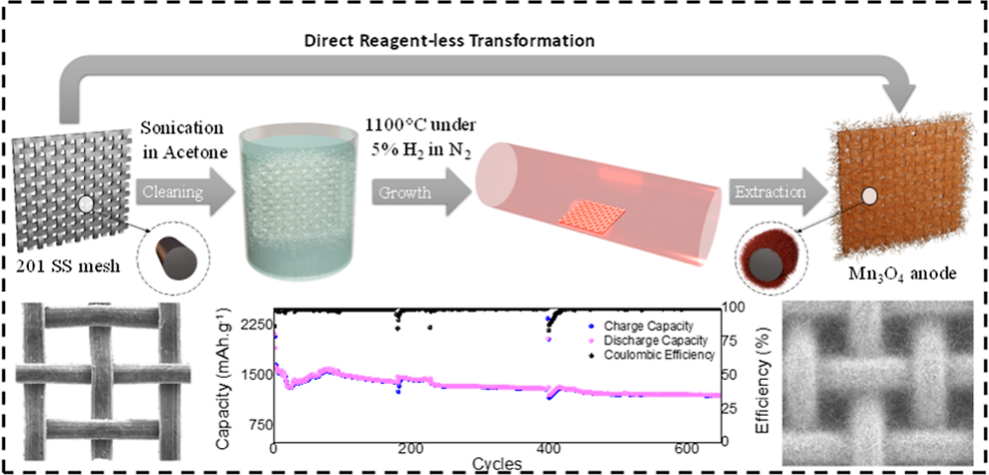

Transition metal oxides (TMOs) have been widely studied as potential next-generation anode materials, owing to their high theoretical gravimetric capacity. However, to date, these anodes syntheses are plagued with time-consuming preparation processes, two-dimensional electrode fabrication, binder requirements, and short operational cycling lives. Here, we present a scalable single-step reagentless process for the synthesis of highly dense Mn_3_O_4_-based nanonetwork anodes based on a simple thermal treatment transformation of low-grade steel substrates. The monolithic solid-state chemical self-transformation of the steel substrate results in a highly dense forest of Mn_3_O_4_ nanowires, which transforms the electrochemically inactive steel substrate into an electrochemically highly active anode. The proposed method, beyond greatly improving the current TMO performance, surpasses state-of-the-art commercial silicon anodes in terms of capacity and stability. The three-dimensional self-standing anode exhibits remarkably high capacities (>1500 mA h/g), a stable cycle life (>650 cycles), high Coulombic efficiencies (>99.5%), fast rate performance (>1.5 C), and high areal capacities (>2.5 mA h/cm^2^). This novel experimental paradigm acts as a milestone for next-generation anode materials in lithium-ion batteries, and pioneers a universal method to transform different kinds of widely available, low-cost, steel substrates into electrochemically active, free-standing anodes and allows for the massive reduction of anode production complexity and costs.

## Introduction

Major advances in technological applications, such as portable or stationary electronic devices and electric transportation, pose a matching demand for energy storage devices with great stability and high energy densities. Since their commercialization, lithium-ion batteries (LIBs) have dominated as the primary choice to fuel these applications. In this context, graphite has been the most common anode material since the introduction of LIBs in 1990, owing to its stability due to minimal structural changes during lithium intercalation. However, graphite has a theoretical gravimetric capacity of 372 mA h/g, which is not in par with the growing demand for higher-energy-density devices. Thus, research on novel candidate materials is constantly conducted to find a replacement for the existing graphite in commercial cells. In the last decade, multiple materials have risen as potential next-generation anode materials, notably lithium-alloying materials such as silicon- and phosphorus-based nanostructured and microstructured materials, owing to their intrinsically high theoretical capacity.^1−6^ In this context, silicon nanowires (NWs) have been the focus of extensive research efforts in a broad range of applications, including their potential as future high-capacity anodes in LIBs.^7−14^ In practice, however, the huge volumetric changes experienced by these materials during the lithiation and delithiation cycles cause a rapid performance degradation. Furthermore, most reported strategies are based on complex, costly, and low-content active material composite anodes, thus limiting their real-world applicability.^15−17^ As such, attempts at commercializing pristine silicon anodes were unsuccessful thus far, which is only possible by embedding silicon into carbon-based matrices. Several C@Si anode materials are already in circulation; however, the low amount of silicon has greatly affected their capacities, which range between 400 and 1000 mA h/g.^18^

In this context, transition metal oxides (TMOs) are another class of materials, widely investigated as potential candidates for replacing graphite as next-generation anodes.^19−23^ TMOs offer a different lithiation mechanism where the diffused lithium ions are converted into Li_2_O *via* a conversion reaction. The multielectron transfer reaction results in the formation of metallic elemental species. Owing to the large number of electrons which participate in the reaction, TMO-based anodes boast high theoretical capacities (between 650 and 1000 mA h/g) all the while experiencing smaller volumetric expansions in comparison to alloying-based materials. Several TMOs have been widely investigated, mainly including iron,^24,25^ cobalt,^26^ copper,^27^ chromium,^28,29^ and titanium oxides.^30,31^ State-of-the-art advances in the field focus on nanostructured TMOs and TMO composites which enhance the rate performance and reversible capacities due to a significant decrease in Li^+^ pathways and volumetric change, such as nanospheres,^32,33^ NWs,^34,35^ and nanotubes.^36^ Despite showing promising results, owing to their high specific capacities, they are crippled by several detrimental factors, such as toxicity, low conductivity, and volumetric changes occurring from lithiation and delithiation events, which cause active delamination from the current collector, rendering the material inactive.^37,38^ In order to accommodate the volumetric changes, the materials should be mixed with inactive materials and exhibit a high porosity of more than 40% for the electrode to be applicable in a full-cell apparatus, which is hardly achievable in the literature using common electrode fabrication processes.^1^ More importantly, most of their preparation routes require the use of binders and conductive additives to adhere the active particles to a current collector and compensate for their lack of conductivity, enforcing a two-dimensional (2D) electrode configuration which reduces the energy density of the final electrode.^39^

In order to overcome the above-mentioned limitations, we herein present the simple and scalable single-step synthesis of a novel, three-dimensional (3D) composite electrode, based on the self-transformation of stainless steel substrates to monolithic Mn_3_O_4_ NW-structured electrodes. While many reports are plagued by time-consuming synthetic routes, the single-step synthesis presented here relies on a simple thermal treatment of the steel substrate, without any reagents, that results in a highly efficient solid-state transformation of steel into a self-grown highly dense forest of Mn_3_O_4_ NWs. The extraordinarily large surface area of the anode, along with its 3D properties, conveys excellent electrochemical performance including remarkably high capacities (∼1500 mA h/g), great cycling stability (>650 cycles), high Coulombic efficiency (>99.5%), and high C-rates (>1.5 C). The work here may provide a paradigm-shifting niche for TMOs, specifically for manganese oxide, due to the many advantages the synthetic route given here provides. As the proposed fabrication relies on a single-step thermal synthesis, most of the cost is directly related to the substrate. The 201 steel costs less than $1300 per ton, which is an order of magnitude less than battery-grade graphite, along with being cheaper than common synthetic methods. Furthermore, the unique 3D architecture features direct growth of NWs, which increases the conductivity of the whole anode, allows for a complete free-standing electrode, and provides stable solid-electrolyte interface (SEI) formation. The extremely low-cost direct transformation of the low-grade steel substrates into electrochemically highly active anodes offers a novel experimental paradigm that allows the universal self-transformation of different grades of steel substrates into low-cost, free-standing, and binder-free highly efficient anode materials.

## Methods

### Anode Fabrication

The self-transformation of the low-grade 201 steel was conducted by placing the as-received 201 steel mesh (120 mesh, 80 μm fiber diameter) in a home-built 4 in. quartz tube oven system. Prior to the oven placement, 10 min of sonication in acetone was conducted in order to remove all possible organic contaminants. The oven was flushed with 200 sccm N_2_ (99.5%) after reaching the base pressure in order to stabilize the chamber pressure to 550 torr. The temperature was then ramped at 20 °C/min to 1100 °C under a combined atmosphere of H_2_ (99.999%, 10 sccm) and N_2_ (99.5%, 200 sccm). In typical experiments, once the temperature has reached the desired set point, the substrate was left under the above-described conditions for up to 12 h before letting the oven cool down to room temperature. The loading of the active material was determined after growth by sonicating a known area of the resulting sample and calculating the weight difference after sonication for 30 min.

### Single NW Device Fabrication

Conductivity measurements of single wires were conducted by directly measuring the conductivity using a two-electrode system. A 3 in. silicon wafer with 600 nm wet thermal oxide was spin-coated with LOR5A at 4000 rpm for 45 s and baked on a hotplate at 180 °C for 5 min. Afterward, AZ1505 was spin-coated at the same speed and baked at 100 °C for 1 min. An outer electrode mask was exposed using UV lithography at 17 mJ/cm^2^ and developed in AZ726 for 1 min, followed by a deionized water rinse. 5 nm Cr and 60 nm Au were evaporated to create the outer electrodes and were placed in N-methyl-2-pyrrolidone (NMP) for lift-off. Once prepared, relevant manganese oxide NWs (after growth, fully lithiated, and after one cycle) were sonicated off the electrode in isopropyl alcohol (IPA) and dripped onto a dye with an outer electrode pattern. Once prepared, electron-beam (e-beam) lithography was conducted in order to place two electrodes on single NWs present on the surface of the silicon dye. LOR5A was spin-coated and baked as previously described. ZEP 520A was spin-coated afterward at 3000 rpm for 40 s and baked at 180 °C on a hotplate for 2 min. Electrodes were exposed using 10 kV and a 20 μm aperture with a dose of 70 μC/cm^2^ and a 0.025 μm step size. 450 nm Ni was then evaporated in order to create the electrode contacts and to fully cover the NWs. The lift-off process took place in NMP. Measurements were conducted between −2 and 2 V at a rate of 0.2 V using a home-built probe state.

### Materials Characterization

Microscopy images were taken using a scanning electron microscope (Quanta 200FEG, Jeol Co.) and a high-resolution scanning electron microscope (Gemini 300, Zeiss). Transmission electron microscopy (TEM) and scanning TEM (STEM) and energy-dispersive X-ray spectroscopy (EDS) analyses were conducted using a Themis Z system. The fast Fourier transform (FFT) was resolved using CrystBox diffractGUI. X-ray photoelectron spectroscopy (XPS) measurements were conducted using a Scanning 5600 AES/XPS multitechnique system (PHI, USA). X-ray diffraction (XRD) measurements were conducted using a Bruker D8 Discover diffractometer.

### Electrochemical Measurements

The prepared composite anodes were punched into discs of 10 mm in diameter and were smeared in a commercial 0.2% single-walled carbon nanotube ink in NMP (OCSiAl) to improve the electrical contact to the current collector. The electrodes were dried in a vacuum oven at 100 °C overnight (approximately 12 h). CR2032 coin cells, containing two spacers, the composite Mn_3_O_4_ anode, a separator, lithium metal, and a spring, were assembled after drying in a glovebox (<0.1 ppm O_2_). The separators were 2325 Celgard separators, and the lithium metal discs (15 mm in diameter) were purchased from S4R, France. 35 μL of 1 M LiPF_6_ in ethyl carbonate (EC)/diethyl carbonate (DEC) (1:1) with 2% vinylene carbonate (VC) as an electrolyte was used in all of the experiments (Solvionic, France). The cells were kept at a constant temperature of 25 °C throughout all the experiments. Electrochemical measurements were conducted using a BioLogic BCS battery cycler. Impedance spectroscopy measurements were conducted using a BioLogic BCS battery cycler from 10 kHz to 0.01 Hz after delithiation. Post-mortem analysis was conducted by disassembling the coin cells in a glovebox and extracting the cycled anode. The anode was either taken as-extracted or was washed with diethyl carbonate (DMC) for 1 h in order to remove the secondary SEI, where specified.

## Results and Discussion

Stainless steel is a widely used material in a myriad of applications as a common building block due to its resilient chemical, electrical, and physical properties. In this context, stainless steel is utilized solely as casing parts, and seldom as current collectors, due to its chemical inertness and high electronic conductivity. Previous studies indicate that stainless steel’s potential can be unlocked by using its endogenous metal species as self-catalyzing agents that allow the growth of different forms of nanomaterials *via* the introduction of external coreagents.^5,6,40^ However, the ability of the steel substrate to act as a self-catalytic bed for the growth of nanomaterials in a “reagentless” manner, strictly consisting of the elements assembling the steel, is hardly investigated.^41^ Successful reagentless synthesis would not only impact the potential future uses of this commonly known, chemically inert material, but may also prove as a universal method of solid-state chemo-morphological transformation of many grades of steel substrates. Stainless steel 316, SS316, is one of the most commonly used stainless steel grades nowadays and was the subjected catalytic bed in previous reports,^41,42^ leading to the formation of low-density MnCr_2_O_4_ spinel nanostructures.

Here, as a proof-of-concept, the low-cost 201-grade steel was used as an economical alternative for 316SS. The differences between the compositions of these two steel grades are shown in Table 1.

**Table 1:** Chemical Composition of 201 and 316 Stainless Steel Types

	Mn (%)	Cr (%)	Ni (%)	C (%)	Si (%)	S (%)	Fe (%)
201 SS	5.5–7.5	16.0–18.0	3.5–5.5	0.15	1.0	0.03	bal.
316L SS	<2.0	16.0–18.0	10.0–14.0	<0.07	1.0	0.03	bal.

As can be seen, the significant change in the steel types arises from the difference in their Mn content over Ni content, which brings a large difference in mechanical and chemical properties as well as a large cost difference. As nickel is a regulated metal, the price differences between Ni and Mn can reach 100-fold in favor of the much cheaper Mn. Successful solid-state thermal self-transformation of the 201-grade steel would not only cause a considerable cost reduction in the synthesis of novel anode materials, but would also greatly impact the universality of the transformation processes and the arsenal of nanomaterial compositions that may be accessible by a simple change in the source steel’s composition.

**Figure 1:**
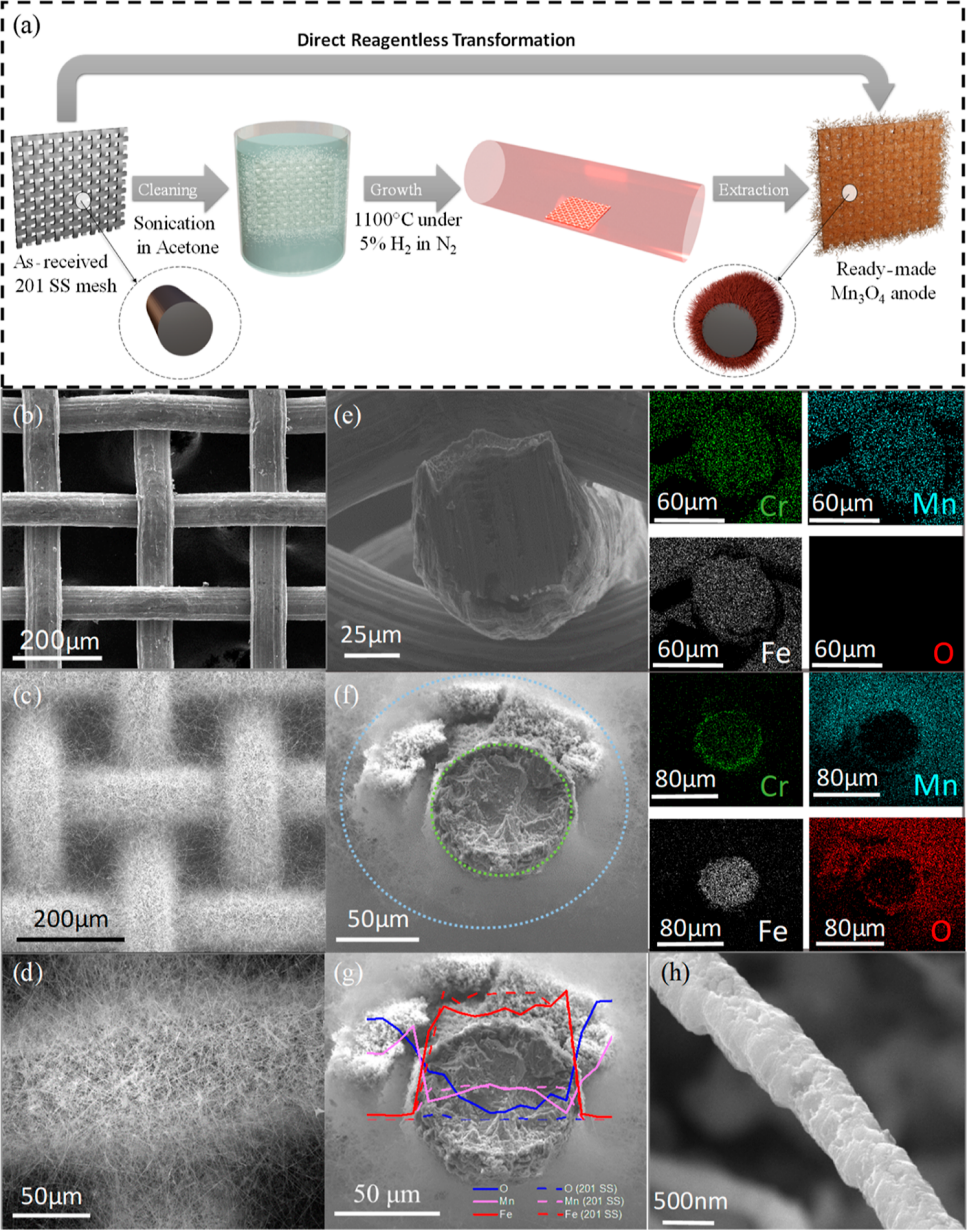
(a) Schematic illustration of the single-step thermal growth process. (b) SEM image of the pristine, as-received 201 stainless steel mesh. (c) SEM image of the stainless steel mesh after 12 h of growth. The dense MnNWs are shown to completely cover the open windows between the fibers. (d) Zoomed-in SEM image on a single fiber after 12 h of growth. (e) SEM image of a cross-sectioned pristine 201 mesh fiber. The right image shows the EDS mapping of the corresponding fiber. (f) SEM image of a cross-sectioned 201 mesh fiber after 12 h of growth. The right image shows the EDS mapping of the corresponding fiber. (g) EDS line scan of a single low-grade 201 steel after growth (full lines). The dashed lines indicate the atomic percentage prior to growth. (h) SEM image of a single typical MnNW, exhibiting a diameter of ca. 500 nm.

The self-transformation of the 201-grade stainless steel substrate into a 3D monolithic composite anode structure is schematically shown in Figure 1a. Throughout the experiments, the as-received 201SS mesh substrate was first sonicated in acetone for 10 min to remove organic contaminants that may be present on the surface of the steel. The washing process is not obligatory but is conducted in order to avoid unwanted carbonization of contamination materials at high temperature, which may affect the self-growth of NW structures. The cleaned sample is then inserted into a tube furnace under vacuum and purged with nitrogen. As the NW synthesis is strictly based on the oxidation of metal species contained in the steel substrate, low-purity nitrogen (99.5%) was used to allow traces of oxygen to be present during the process (oxygen levels were in the range of 5000 ppm or less). The chamber temperature is then ramped to 1100 °C at a rate of 20 °C/min with the flow of H_2_ gas that is introduced into the chamber to create an atmosphere of 5% H_2_ in N_2_ (typically 10 and 200 sccm, respectively) at a total pressure of 550 torr. At the end of the transformation process, the chamber is allowed to naturally cool down to room temperature. Figure 1b shows a pristine, as-received 201 stainless steel mesh substrate. No visible defects can be seen on the steel surface. In the presented case, the steel mesh is composed of 80 μm diameter fibers with a 130 μm window opening. Figure 1c,d shows a typical, thermally transformed, NW-structured steel substrate after 12 h of growth. The NWs are shown to grow directly from the steel’s surface, possessing an extremely long structure, completely covering the open window space of the mesh structure, with NW lengths reaching more than 130 μm. Figure 1d, which presents a zoomed-in image of a single, thermally transformed, steel fiber, shows the formation of an extremely dense and uniform NW-based matrix, covering the entire surface of the steel fibers. The ability to grow extremely long and uniform NWs, strictly by thermally treating the steel substrates by this very simple and cost-effective approach, allows for a simplistic control over the porosity of the resulting electrode and hints at the high active material loadings that can be achieved, reaching values of 3 mg/cm^2^. In order to gather insights regarding the plausible NW growth mechanism, EDS was performed at the cross-section of the steel fibers. Figure 1e shows a scanning electron microscopy (SEM) image of a cross-sectioned pristine stainless steel fiber. Mapping of the abundant elements of interest (Fe, Cr, Mn, and O) shows an even distribution along the bulk volume of the fiber, without any oxygen present in the elemental mapping. Although oxygen is not detected in the elemental mapping, some surface traces of oxygen can be found due to exposure of the otherwise unexposed steel surface during the cross-section preparation. Therefore, the atomic percentages found on the surface may slightly differ from those in the composition shown in Table 1. After the thermal-transformation growth, as shown in Figure 1f, the elemental composition and morphology of the steel fibers is completely altered. The green and blue encircled areas are used to differentiate between the steel core fiber and the resulting covering NW matrix, respectively. Post-growth, oxygen is present as a key element, found primarily on the surface of the steel fiber core and as a component of the NW matrix. Mn is seen to almost completely diffuse outward from the steel core, hinting the formation of manganese oxide NWs. Cr is also seen to diffuse toward the perimeter of the steel fibers, while Fe strictly remains at the core section. Small traces of both elements can be detected in the NW-based region. The elemental composition shown in Table S1 confirms the major changes occurring at the steel fiber surface, where Mn and O are the dominating elements in the synthesized forest-like nanostructured steel. Further line scan characterization elucidates the potential of the nanostructuring of steel. Figure 1g shows a line scan of the 80 μm low-grade steel fiber post-transformation. The lines demonstrate the atomic percentage state of Fe, Mn, and O outside and inside the fiber perimeter. As can be visualized from the spectra after growth (bold lines), both Mn and O are highly concentrated in the NW region, while their concentration drops toward the fiber’s core. In contrast, the concentration of Fe remains high only at the core of the fiber. As can be seen prior to the growth (dashed lines), Mn and Fe concentrations are constant along the entire fiber, without O. The line spectra of the different elements allow for a deeper understanding of the effect of the low-grade steel’s core fiber diameter. As the Mn concentration in the fiber’s core remains approximately similar prior and post-growth, it implies that by using a thinner mesh, higher loadings can be achieved as more of the Mn element will be available for oxidation under the same conditions. Even with the selected mesh, the number of grown NWs is unprecedented. The ability to further improve the resulting loading by lowering the diameter of the core fibers will also improve the areal and volumetric capacities, along with lowering the overall weight of the anode. As evidence, Table S2 lists the amount, in mg/cm^2^, of the elements composing the 201 steel. By determining a 3% (by weight) growth of Mn_3_O_4_, it can be seen that only one-third of the Mn content has been used, which corresponds to approximately 9% (by weight) maximum theoretical loading under optimal conditions. Figure 1h shows a SEM image of a single NW, with a diameter of ca. 500 nm. It should be noted that the NWs grown exhibit a large range of sizes, from 50 to 500 nm, with most NWs displaying an average diameter of ca. 300 nm, all composed of the same material. As can be seen from these images and the elemental composition post-growth, the NWs grow directly from the surface of the steel fibers, without any external reagents introduced to the reaction. Supporting Information Figure S1 shows a MnO NW structure grown for 20 min, with a small particle at the top, with its corresponding EDS measurements given in Table S1. The spectra confirm that the particle is composed of large amounts of Fe, indicating that the particle is a part of the underlying steel fiber being pushed outward as the NWs grow. It is noteworthy to mention that the NW exhibits roughly the same diameter as the NW in Figure 1g, which indicates that longer growth times affect the overall length of the resulting NWs rather than their diameter, thus increasing the aspect ratio of each wire and greatly increasing the surface area of the as-synthesized electrode.

Past investigations regarding the change in elemental composition of stainless steel during heat treatment have resulted in the realization of the different oxidation energies of each element. In terms of the main abundant elements and the elements of interest, Mn exhibits the highest affinity toward oxidation, followed by Cr, Ni, and Fe.^43,44^ The corresponding thermodynamically stable oxides, with the lowest energies of formation, are MnO, Cr_2_O_3_, NiO, and Fe_2_O_3_, respectively. As Mn is evidently found at high concentrations on the surface of the 201 steel fibers, once oxidized, MnO covers most of the steel’s surface.^45^ The reducing atmosphere allows further distinction between the oxidized species, committing available oxygen to further react with the remaining nonoxidized Mn available. As MnO is formed, further oxidation leads to the formation of Mn_3_O_4_,^46^ which composes the NWs in the resulting electrodes. The formation of Mn_3_O_4_ over other manganese oxide types is favorable, as can be learnt from thermodynamic data taken from Ellingham diagrams.^47^ At the presented temperature of 1100 °C, with the amount of oxygen provided in the system, it is apparent that Mn will oxidize prior to other metallic elements in the steel, and Mn_3_O_4_ will be created. Supporting Information Figure S2 shows SEM images that shed light on the apparent mechanism of the NW growth. While ramping the system to 1100 °C without oxygen, rearrangement of the steel’s surface can be noticed, with the creation of nano-sized domains. When ramping with oxygen available in the system, elongated structures begin to form, composed solely of Mn_3_O_4_, while chromium remains on the surface. Therefore, we hypothesize that the NWs, composed of crystal pallets that rise from the surface, as Mn continuously oxidizes, grow from physical confinement, occurring as the surface of the steel rearranges during the ramping to high temperature.

**Figure 2:**
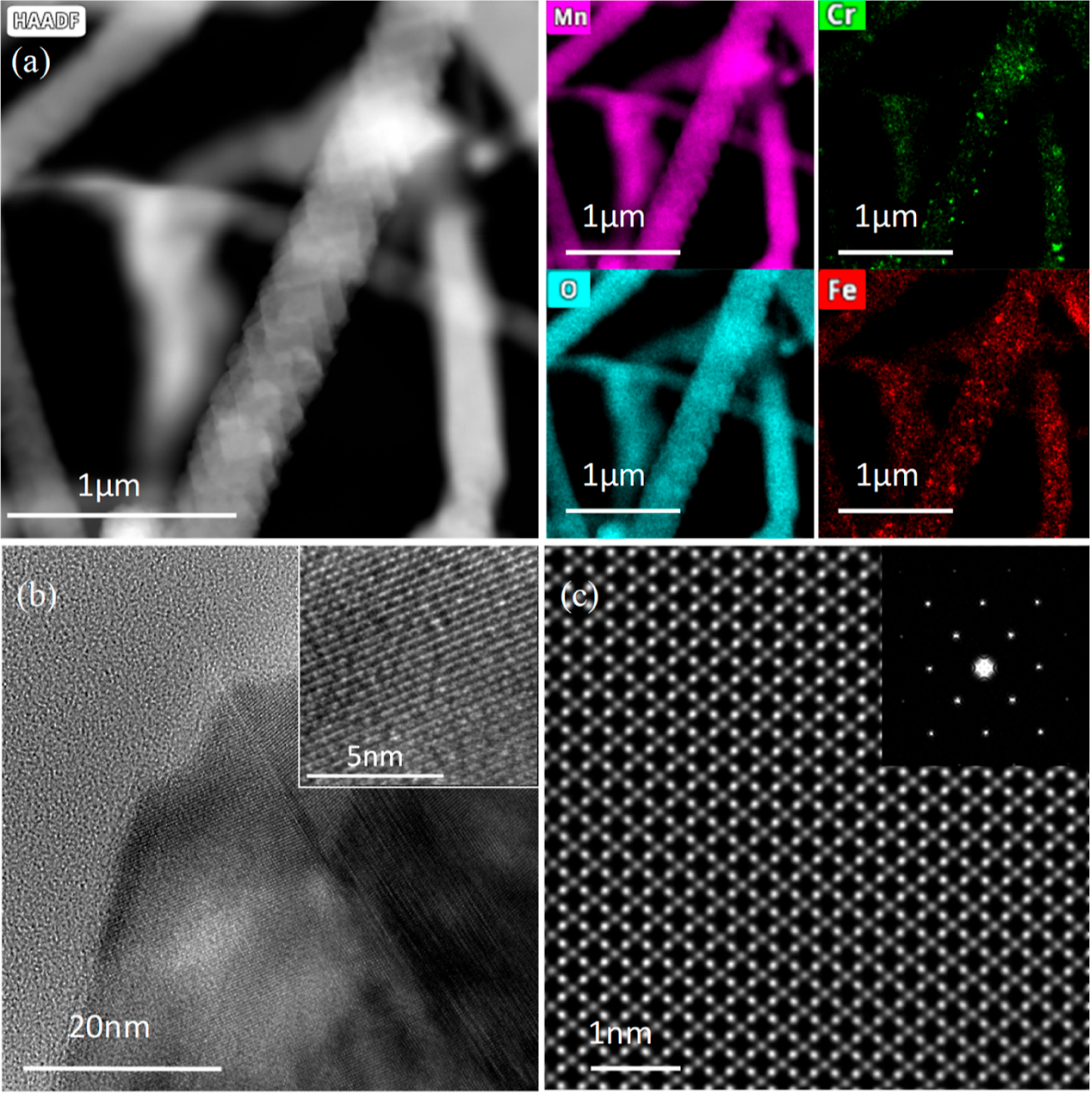
TEM analysis of the resulting Mn(CrFe)NWs. (a) STEM micrograph of a single NW exhibiting a layered growth pattern. The right images are the EDS mappings of the NW, showing high concentrations of Mn and O, with spread inclusions of Cr and Fe. (b) High-resolution TEM image of the twinning defect and the layered growth. Inset: High-resolution image of the wire, showing the highly crystalline structure of the NW. (c) High-resolution STEM image of an area in (c). Single-crystal formation can be seen. Inset: corresponding diffraction pattern of the same area.

TEM image of a single NW is provided in Figure 2. Figure 2a shows a high-angular annular dark-field (HAADF) image of a single NW, exhibiting a diameter of ca. 300 nm. EDS analysis clearly shows the abundance of Mn and O elements composing the NW and spread uniformly across it. Furthermore, Cr and Fe elements are also present in the form of defined inclusions within the matrix of the NW. The direct growth of the NWs from the surface of the steel allows for some diffusion of contaminants, such as Fe and Cr, to be incorporated in the multielemental growing matrix due to the high temperatures of the growth process. Notably, the NW is shown to be composed of stacked layers, resembling a growth with twin defects. Figure 2b shows a high-resolution TEM image at the border between two plates, which build the NW throughout its length. It can be seen that the NW is highly crystalline (Figure 2b, inset). Figure 2c shows a high-resolution STEM image at the ⟨110⟩ zone axis, further confirming that the composition of the NW is Mn_3_O_4_.

**Figure 3:**
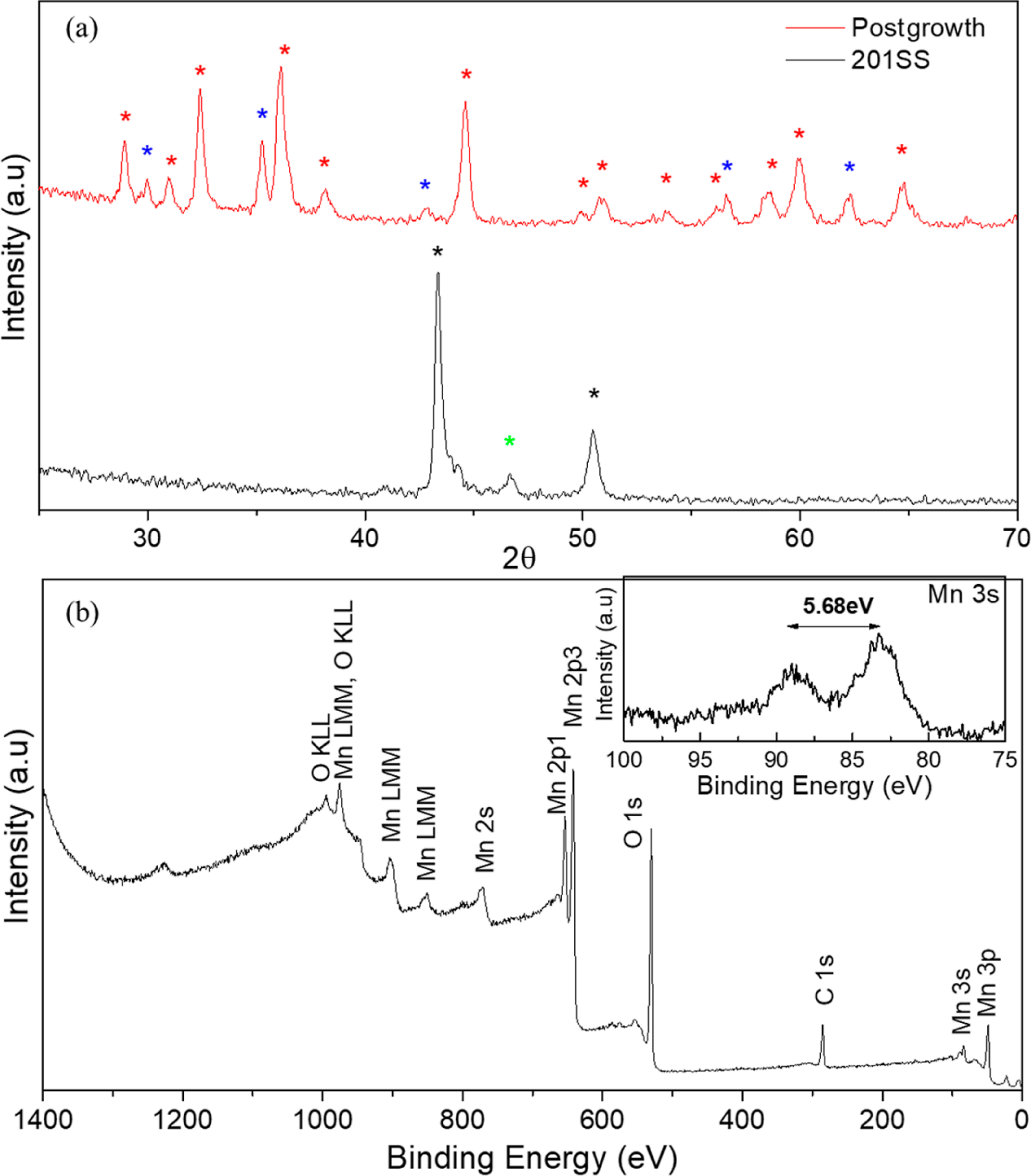
(a) XRD analysis of the pristine 201 stainless steel mesh (black line) and after 12 h of growth (red line). The black asterisks mark the austenitic phase, the green asterisk marks a CrMn_3_ phase, the red asterisks mark the Mn_3_O_4_ phase, and the blue asterisks mark the Cr_1.807_Mn_1.193_O_4_ phase. (b) XPS spectrum of a thermally transformed 201 stainless steel substrate. The inset shows a high-resolution measurement of the Mn 3s peak, exhibiting a signal split corresponding to the Mn_3_O_4_ phase.

Further characterization of the Mn(CrFe)NWs is shown in Figure 3. XRD analysis of the pristine, as-received 201 stainless steel (black line) and after 12 h of growth (red line), shown in Figure 3a, demonstrates a clear morphological difference. The pristine steel substrate exhibits austenitic phase peaks (marked with black asterisks) located at 43 and 50° (PDF 00-033-0397) and a small peak which may correspond to a chromium–manganese alloy (marked with green asterisk), located in 46° (PDF 04-004-8453), due to the high manganese content in steel. After being subjected to 12 h of a semireducing atmosphere, the XRD analysis reveals a different result. The peaks which formerly coincided with the austenitic phase of the steel disappeared. The peaks located at 29, 31, 33, 36, 38, 45, 50, 51, 54, 56, 58, 60, and 65° (marked with red asterisks, PDF 01-086-3860) are associated with Mn_3_O_4_ crystalline phases, further confirming the data concluded from the TEM analysis. The other peaks, marked with blue asterisks and located at 30, 35, 43, 57, and 62°, are associated with a Cr_1.807_Mn_1.193_O_4_ phase (PDF 04-023-4174). This phase can be associated with the outer oxide layer that is formed during the growth process along the perimeter of the stainless steel fibers, matching the data received from the mapping provided in Figure 1f. XPS analysis was performed in order to confirm the oxidation states of the Mn species. Figure 3b shows a survey XPS spectrum of a steel substrate after 12 h of growth. In order to verify the oxidation states of Mn, high-resolution measurements were conducted to properly view the Mn 3s orbital peak. Due to the coupling of nonionized 3s electrons with 3d valence electrons, the broadening in the peak splitting of Mn 3s can be used to differentiate between the different oxides.^48^ Although Mn 2p (Supporting Information, Figure S3) can be used to characterize manganese oxides, it is difficult to distinguish between the different oxides. Therefore, high-resolution XPS is used to view Mn 3s properly. As shown in the inset, it is possible to deduce the oxidation states by measuring the signal split and comparing it to theoretical values, which correspond to a signal split of 5.5 eV and 5.9 eV between the two peaks for Mn^3+^ and Mn^2+^, respectively. Here, the signal split value is measured to be 5.68 eV, corresponding to mixed oxidation states, common to the Mn_3_O_4_ spinel phase.

**Figure 4:**
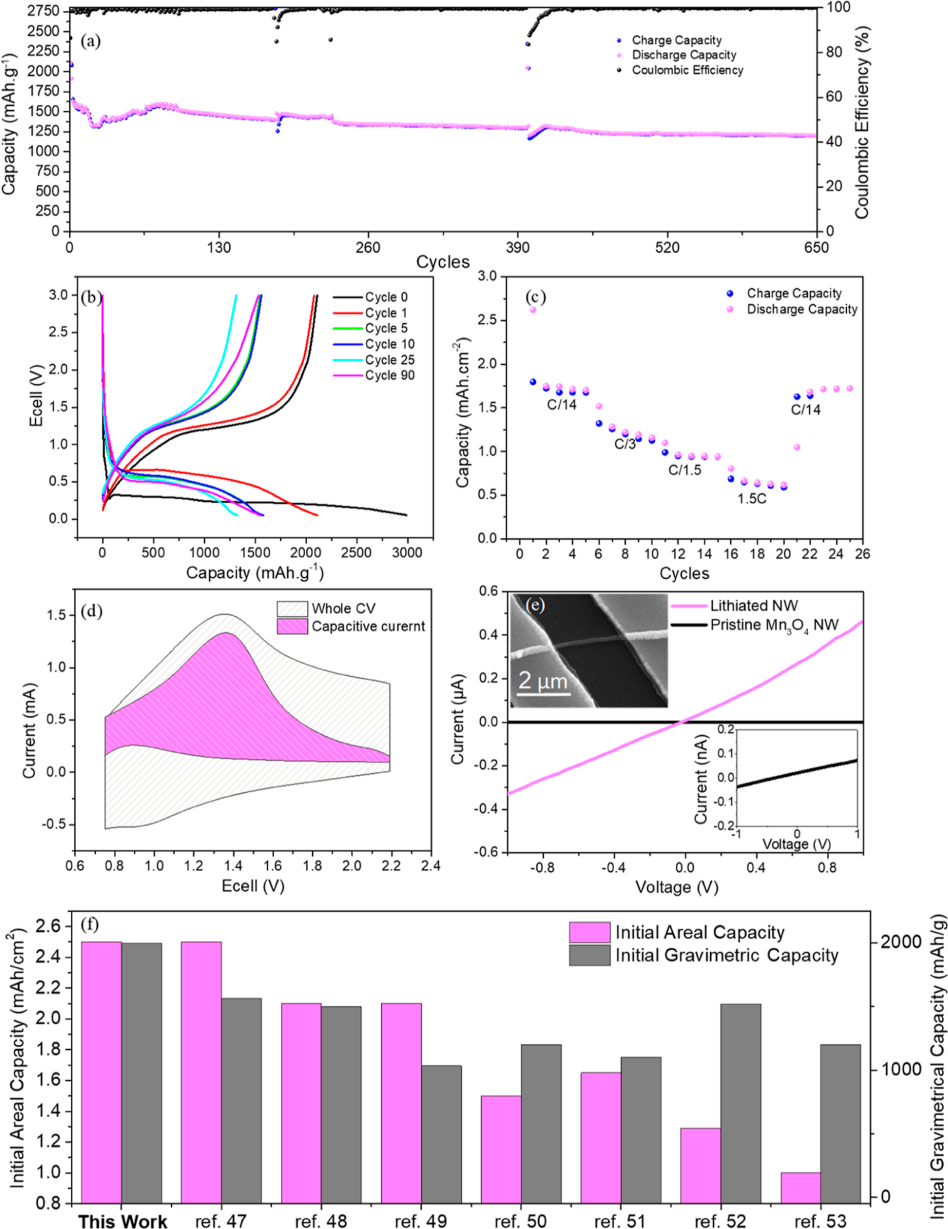
Electrochemical performance of the MnNW anode. (a) Cycle life of a typical cell, cycled at 500 mA/g (C/4) between 0.05 and 3 V, exhibiting a stable cycle life over 650 cycles. (b) Voltage profile of the cell depicted in (a). (c) C-rate performance of the transformed stainless steel anode. (d) CV analyzed with the Dunn method to deduce the capacitive current from the overall current at 3 mV/s. (e) Conductivity measurements of pristine and fully lithiated MnNWs. Inset top: typical device created in e-beam lithography. Inset bottom: zoomed-in view of the current resulting from the pristine MnNW. (f) Comparative scheme of the present work with various recent publications of TMO-based anodes in terms of areal and gravimetric capacities. This work exhibits a superior performance in comparison to recently published works in both aspects.

Electrochemical characterization of the composite electrodes was conducted in order to assess the performance of the material as a potential anode in LIB applications. The monolithic, free-standing, composite anode substrates were cut into 10 mm discs and used as electrodes versus lithium metal in a 2032-coin cell configuration. All the cells were cycled between 0.05 and 3 V. Figure 4a shows the electrochemical performance of a typical anode. The gravimetric capacity was calculated by considering the direct mass of NWs formed on the steel substrate. The cell was cycled at 500 mA/g, corresponding to approximately C/4 by taking into account the practical reversible initial capacity. The first two cycles were cycled at C/30 in order to assess the overall capacity of the cell. The initial drop in capacity, related to the irreversible loss in capacity, is calculated to be 29.6%. This drop is caused due to the primary formation of the SEI on the 3D electrode, along with irreversible changes occurring in the Mn_3_O_4_ NWs due to the conversion reaction and lithiation mechanism. After the initial drop and upon faster cycling, the cell capacity remains remarkably stable for more than 650 cycles, demonstrating an impressive capacity retention (measured from the first cycle of the C/4 rate) of 74%, with a capacity > 1200 mA h/g. The overall Coulombic efficiency of the cell is calculated to be 100%. Notably, while performing slow cycling (C/30) approximately every 200 cycles, the amount of the remaining active material can be assessed as kinetic effects are reduced. After 450 cycles, a 22% capacity reduction is observed from the initial capacity, indicating that the delamination rate of the NWs from the core fibers is low. Figure 4b shows the voltage profile of the same cell, showing a slight capacity decrease over the ongoing cycles. In order to further assess the performance of the potential anode, C-rate measurements were conducted. The loading of the active material was calculated by fully sonicating representative pieces from each growth for 1 h in IPA and measuring the weight before and after sonication (which accounts only for the active NW mass). This resulted in a steady 3% change in weight, corresponding to 1.12 mg/cm^2^ of the active Mn_3_O_4_ NW material. Figure 4c shows the rate performance of a typical cell. It can be seen that even under fast cycling conditions, the anode material still exhibits high capacity values. The area specific capacity was calculated by using the area of the anode (0.78 cm^2^). Supporting Information Figure S4 shows a cyclic voltammetry (CV) measurement taken for a fresh cell after a preliminary cycle. The cathodic peak at ∼0.31 V can be associated with the lithiation of the Mn_3_O_4_ NWs, along with the conversion reaction happening toward the formation of Li_2_O. The anodic peak at ∼1.36 V can be associated with the reversible reaction and oxidation of the metallic species created by the conversion reaction, along with delithiation of the entire electrode.^49,50^ The lithiation mechanism proposed in previous studies, along with the mechanism proposed here, is given in eq 1
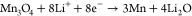
1

In this reaction, eight electrons are transferred, and the theoretical capacity can be calculated as 937 mA h/g for the pure spinel phase.

To investigate the lithiation behavior of the nanostructured anode material, CV under different rates was conducted, as shown in Figure S5. As the active material size decreases to the nano regime, such as in the presented Mn_3_O_4_ NWs, two types of effects may affect the overall capacity extracted from the cell. Along with the Li^+^ insertion, pseudocapacity arising from charge transfer processes with surface atoms along with non-Faradic contributions from the double layer effect may greatly affect the capacity measured due to the highly increased surface area of the active material. Using the Dunn method in eq 2(51)
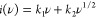
2

we can distinguish between the capacitive-controlled and diffusion-controlled processes by plotting the current received at a fixed potential (*i*) against the different scan rates (ν). This analysis can provide the ratio between the capacitive current (*k*_1_) and the diffusion-controlled current (*k*_2_). From the analysis, it can be seen that even at high cycling rates, such as the one shown in Figure 4d at 3 mV/s, most of the current arises from diffusion-based processes. Figure S6 in the Supporting Information shows that as the scan rate increases, the capacitive contributions increase. At 3 mV/s, the capacitive contribution from the overall current is valued at 45%. Figure S7 shows a linear relationship between log(*i*) and log(ν) extracted from different scan rates at 0.9 V. The slope, calculated to be 0.76, indicates a combined contribution of capacitive and diffusion-controlled processes, which can relate to the SEI formation. In order to extract the diffusion coefficient of Li in Mn_3_O_4_, a galvanostatic intermittent titration technique (GITT) was performed. Using eq 3, the diffusion coefficient can be extracted
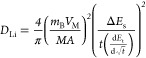
3where *m*_B_ is the loading of the active material, *V*_M_ is the molar volume of Si, *M* is the molecular weight, *A* is the area of the electrode, *t* is the duration time, Δ*E*_s_ is the difference in voltage during the steady state (rest), and *E*_t_ is the voltage in the constant current phase. The measurement was performed by applying a current at a rate of C/15 for 15 min, followed by a rest period of 30 min. Supporting Information Figure S8 shows the corresponding measurement. At the lithiation stage, approximately matching the value of the cathodic peak presented in the CV curve given in the Supporting Information, the diffusion coefficient was calculated to be 9.22 × 10^–13^ cm^2^/s, matching previous reports of lithium diffusion coefficients in Mn_3_O_4_-based nanostructures.^52^ For the delithiation process, the diffusion coefficient of lithium is calculated to be 1.83 × 10^–13^ cm^2^/s, at a voltage of 1.5 V, matching the anodic peak in the CV curve.

To further validate the lithiation mechanism and to measure the electrical conductivity of the resulting NWs, we have conducted a series of conductivity measurements of single NWs. The NWs were sonicated from the steel mesh and dripped into a clean 600 nm silicon oxide/silicon dye, and electrodes were fabricated using e-beam lithography. Figure 4e shows typical current measurements as a function of changing source voltages, between −1 and 1 V, of pristine (red) and lithiated (black) NWs. The pristine Mn_3_O_4_ NWs exhibit extremely low currents, corresponding to a conductivity value of 3.38 × 10^–7^ S/cm. However, the lithiated NWs exhibit an increase of 6 orders of magnitude in their conductivity, with a value of 2.74 × 10^–2^ S/cm. The increase in conductivity can be explained by the formation of metallic Mn, over the former oxidized Mn, which drastically impacts the conductivity of the NWs. Once the NWs have been delithiated, the conductivity is again decreased to a value of 1.59 × 10^–8^ S/cm, which is still higher than the theoretical MnO conductivity value,^53^ which implies the incomplete transformation of Mn_3_O_4_ to MnO. Contrary to 2D TMO-based anodes, these measurements hint toward the possibility of allowing high delithiation currents, where this effect is amplified also for the lithiation current (as evident from the capacities received at different C-rates) due to the direct contact of the NW elements to the stainless steel mesh that serves as the current collector. This allows for an efficient electron transfer from the steel current collector to the NWs and vice versa.

Beyond the electrochemical results presented here, few additional important issues should be noted. First, the simple transformation of stainless steel to a monolithic 3D highly efficient free-standing electrode creates a complete double-sided composite anode material. Second, in terms of areal capacity, a 3% Mn_3_O_4_ NWs mass, as seen in Figure 4a, exhibits a high value of 2.5 mA h/cm^2^. A simple thermal treatment of the steel, without any external reagents, provides high areal capacity which is hardly achieved in the reported literature. Moreover, the resulting areal capacity surpasses many of state-of-the-art commercial silicon anodes due to the need of using low silicon loading in order to compensate for the lack of cycling stability, even in commercial batteries. Notably, the electrochemical properties can be significantly improved simply by changing the stainless steel mesh substrate. While the normalized capacity per active material is high, the normalized capacity which accounts for the whole anode’s weight (26 mg/cm^2^) is calculated to be approximately 100 mA h/g_anode_. In the present case, a 201-type mesh with an 80 μm fiber diameter was used. These parameters imply that the volumetric capacity, considering 2.5 mA h/cm^2^ and a 1 × 1 cm^2^ electrode, can be calculated to be approximately 160 mA h/cm^3^ (considering a 160 μm thickness from two 80 μm fibers). As the NWs themselves grow in random orientations and most of them fill the holes in the mesh structure, they do not significantly add to the total volume of the electrode. By reducing the fiber diameter, the volumetric capacity can be tremendously increased. Notably, reducing the fiber diameter and allowing low mesh properties (big apertures between fibers), allow most of the surface area of the electrode to be filled with the active NW material, dramatically increasing the efficiency of the electrode in terms of volume, area, and capacity per weight. Even by using the dimensions of common 316 meshes, as shown in previous papers^40−42^ (25 μm fiber diameters), the electrodes weigh less than half (approximately 12 mg/cm^2^) and have double the capacity-per-weight of an anode. This does not account for the proposed improvement in the loading from lowering the fiber diameter, which would enable better depletion of Mn from the fiber, resulting in higher active material loadings (as can be seen in Figure 1g where Mn does not fully deplete). Furthermore, by changing the steel type from 201 to steel with a higher manganese content (such as 205), higher active material loadings may be achieved while reducing the cost of the substrate even further. The ability to simply transform the intrinsically inactive steel substrate into a self-standing monolithic active anode structure provides a new experimental paradigm, which may have great effects over the electrochemical properties just through alterations to the substrate itself. Figure 4f shows a comparison of various recent reports on the areal and gravimetric capacity of different TMO-based anodes.^54−60^ This comparison shows the high capacities received in this work as a result of the 3D monolithic, binder-free anode synthesized *via* the direct transformation of the low-grade 201 steel. Moreover, this work exhibits the best ratio between the areal capacity and the active material loading, which can be extrapolated from the capacity ratio.

**Figure 5:**
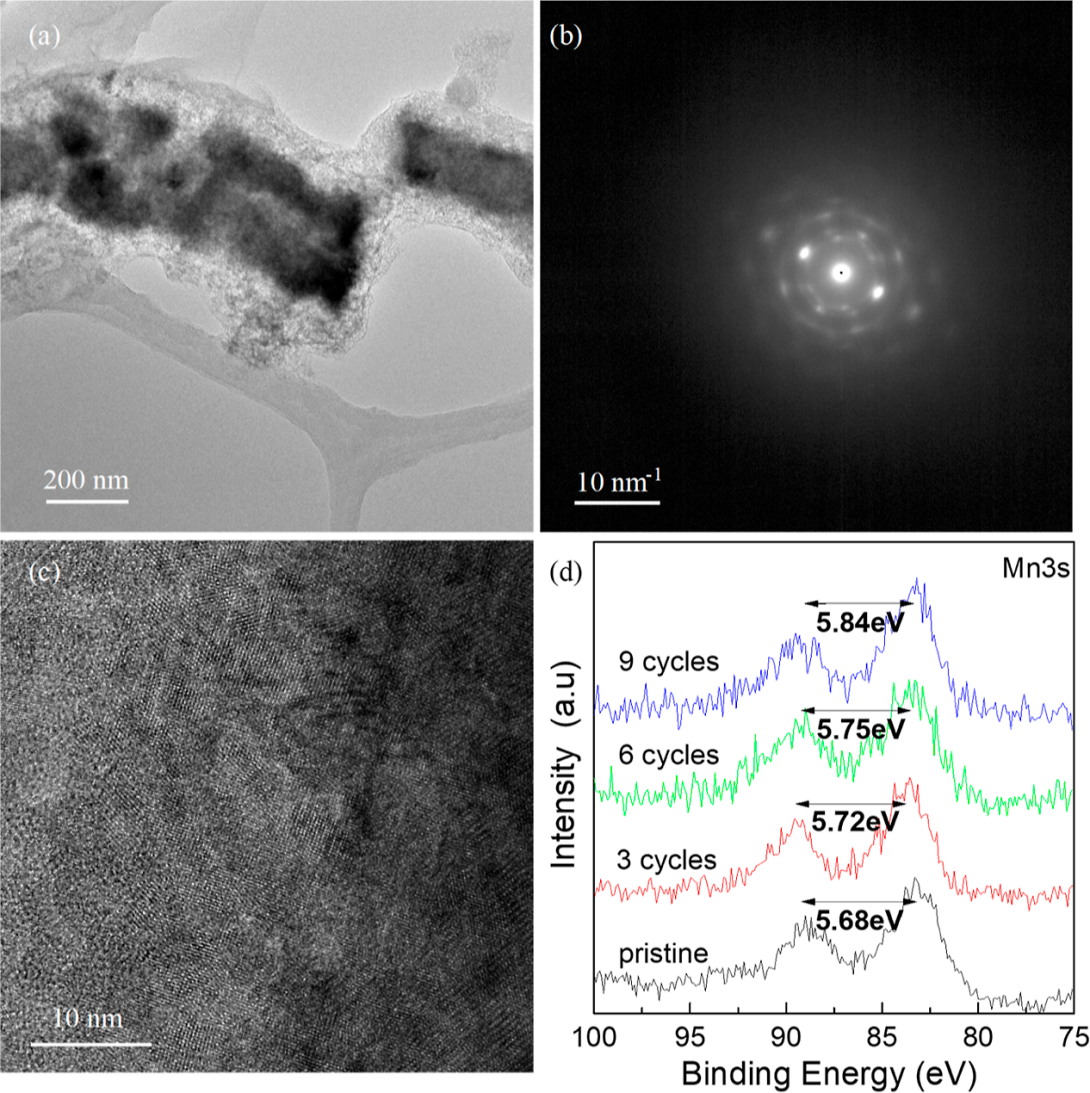
(a) TEM images of a NW after nine cycles, showcasing the amorphous primary SEI layer and the inner NW core; (b) diffraction pattern of the inner part, showcasing the transition to a polycrystalline structure and traces of the amorphous outer layer; (c) HRTEM image of the inner core, showing its polycrystalline structure; and (d) High-resolution XPS spectra of Mn 3s, presenting the rapid transition of the active material from spinel (mixed Mn^2+^/Mn^3+^) to mainly Mn^2+^-based NW oxide over nine cycles.

Furthermore, post-mortem analysis was conducted in order to understand the changes occurring during cycling of the anodes. Figure 5a shows a TEM image of a NW after nine charge–discharge cycles. The anode was washed in DMC for 1 h in order to remove the secondary SEI that was formed during cycling. As can be seen in the image, a core–shell structure is observed consisting of an amorphous SEI shell and an inner oxide NW core. Diffraction patterns at the inner core reveal a polycrystalline structure forming due to the lithiation reaction, as shown in Figure 5b. The smearing of the pattern indicates polycrystalline structures. This is further observed in the high-resolution TEM image (Figure 5c), showing different polycrystalline domains. To further understand the lithiation mechanism and the change occurring in the active material, XPS measurements were conducted at different stages of the anode’s life, from pristine to the ninth cycle. By measuring the split in the Mn 3s signal, the oxidation state of the Mn centers can be deduced and compared to theoretical values. Figure 5d shows an increasing trend in the signal split value, advancing from 5.68 eV in the pristine state toward 5.72, 5.75, and 5.84 eV in the third, sixth, and ninth cycles, respectively. As the value rises, the ratio can be correlated to a shift in the mixed structure and the ratio of Mn^2+^/Mn^3+^, transforming mainly to a Mn^2+^-based material. These measurements conclude the gradual evolution of the pristine Mn_3_O_4_ NWs toward a MnO-based NW structure, which can support the lithiation mechanism described in eq 4(61,62)
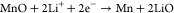
4

**Figure 6:**
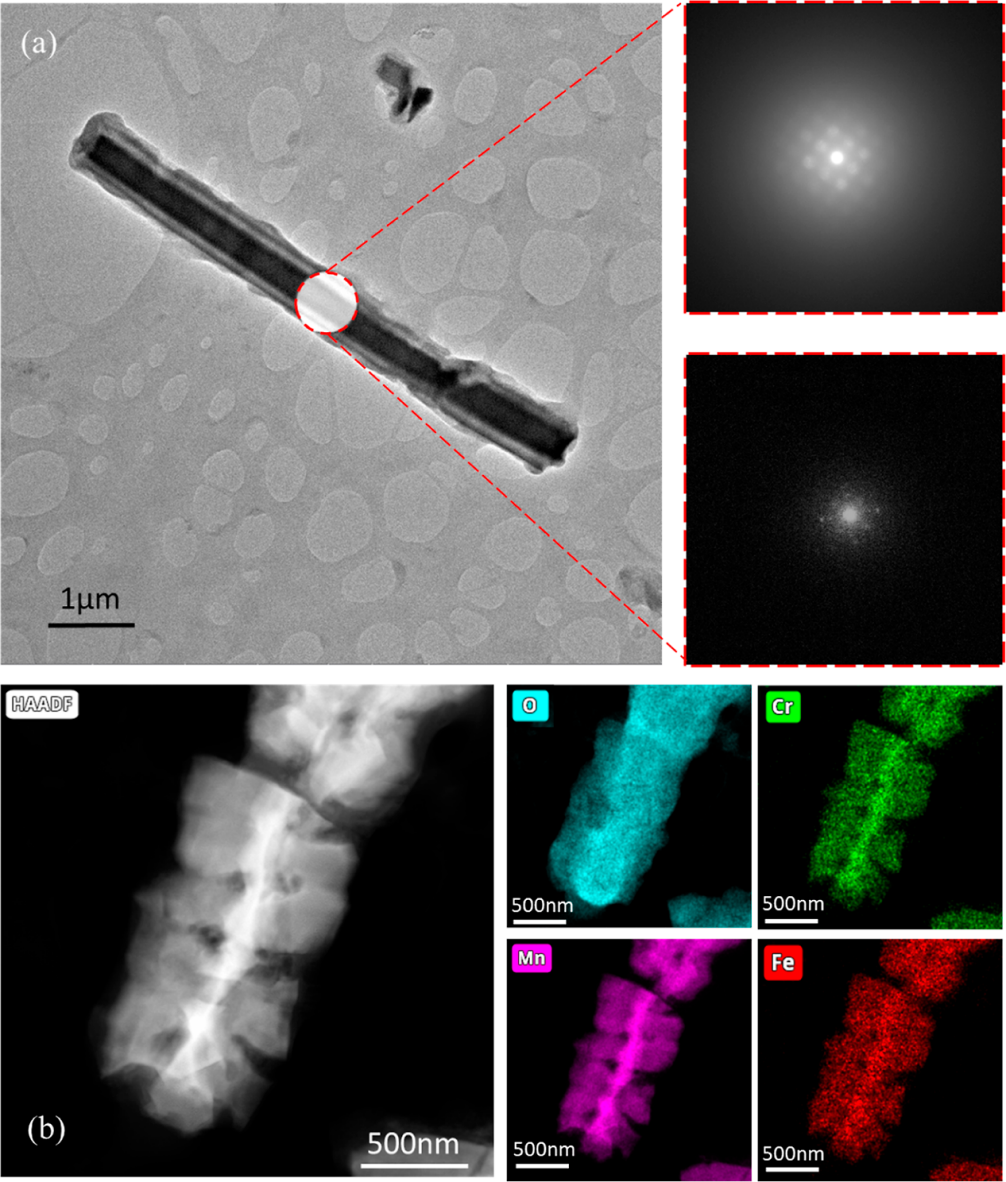
(a) TEM images of a NW after 100 cycles, showcasing a similar core–shell structure consisting of the oxide layer and the primary SEI layer, respectively. The top and bottom insets show the FFT analysis and diffraction pattern of the material, respectively, with clear signs of polycrystallinity. (b) STEM image and EDS mapping analysis of a NW after 100 cycles, showing a porous NW structure.

Long-term post-mortem analysis is shown in Figure 6. Figure 6a shows a TEM image of a NW after 100 cycles. The core–shell structure is still present, as seen previously in Figure 5a. Diffraction patterns reveal that the NW, even after 100 cycles, still remains crystalline, albeit completely polycrystalline, in accordance with the previous analysis. This can shed light on the excellent stability and structural integrity of the NWs formed by the thermal transformation method. Figure 6b shows an STEM image and EDS analysis of a NW after 100 cycles. As can be seen, some of the NWs seem porous, which may positively affect the rate of lithiation kinetics by allowing Li-ions better access to the active material. Furthermore, the evolution of a porous structure may reduce the mechanical stress due to volumetric changes as the porous structure better accommodates the occurring volumetric changes.

Although the literature extensively reports the use of different manganese oxide structures as anode materials, most reports are plagued by time-consuming synthetic routes and binder requirements. Most synthetic routes require a rigorous multistep process, which in the end brings to low areal and volumetric capacities, with a lack of long-term stability. One of the main handicapping properties of modern TMO-based electrodes is the 2D structural configuration that limits the amount of the active material. Furthermore, when dealing with manganese oxide-based electrodes, many methods are handicapped by the lack of ability to create a single material, rather than mixed oxides. Thus, these strategies have not found the proper niche to replace the commercial graphite anode, which exhibits higher electronic conductivity, lesser volumetric changes, higher volumetric capacities, and long-term stability. Here, by using the proposed thermal monolithic transformation of stainless steel, many of the aforementioned properties can be achieved. First, the cost and complexity of preparation of the monolithic anode are reduced by at least an order of magnitude in comparison to that of graphite as 201-grade stainless steel costs less than 1300$ per ton compared to battery-grade graphite which may cost up to 20 000$ per ton.^63^ By changing the steel grade to higher manganese contents, the price can be reduced even further. Second, the synthesized free-standing electrode does not require the use of binders or conductive additives in order to act as a functioning anode. Third, simple modulation of the stainless steel substrate geometry can drastically enhance the resulting electrochemical properties of the anodes. Fourth, the direct growth of the NWs from the substrate highly impacts the mechanical and electrical stability of the whole electrode structure, allowing for hundreds of cycles to be achieved without any severe physical degradation. Furthermore, the electronic conductivity of the active nanomaterial is increased due to the direct, efficient electron transfer from the underlying substrate. Lastly, all the benefits of a 3D substrate, including high active material loadings without concomitant total volume increase, high surface area, stable SEI formation, and the ability to withstand large volumetric changes, are all inherently a part of this simple proposed process. This novel experimental paradigm demonstrates the universality of the proposed process, which yet allows prolific electrochemical results and novel materials to be fabricated by the same, extremely simple, reagentless process of thermal transformation of steel substrates.

## Conclusions

In conclusion, we have presented a novel experimental paradigm that can drastically alter electrochemically inactive 201 stainless steel substrates into a monolithic highly dense NW-structured, free-standing anode structure. These novel anodes, based on self-grown Mn_3_O_4_ NWs from steel’s core fibers, are prepared *via* a single-step thermal treatment without the requirement for external reagents, based solely on differences in the oxidation energies of the inherent metal species composing the steel substrate. The manganese oxide NW-based resulting anodes exhibit excellent electrochemical properties, high capacities and stability (>1200 mA h/g after 650 cycles), high Coulombic efficiencies (100%), and fast C-rates (>1.5 C). The free-standing anode preparation requires no binders and conductive additives as the direct growth of the NWs bestows efficient electron transport from the stainless steel mesh substrate. Single NW conductivity measurements, along with several characterization techniques, have provided insights regarding the lithiation mechanism of the NWs. This work demonstrates a universal, extremely simple, and cost-effective experimental paradigm to transform inactive stainless steel substrates into highly active electrode materials, providing a key step forward in the route toward replacing commercial graphite anodes.

## Supporting Information Available

Elemental composition of steel at different stages of growth, weight percentage calculations of the active material, SEM images of a NW’s tip and the steel’s surface after ramping to a high temperature in different atmospheres, high-resolution XPS spectrum of Mn 2p, cyclic voltammograms at different scan rates, capacitive contribution calculations, and GITT analysis (PDF)
