# Supplementary material for: Three-Dimensional Monolithically Self-Grown Metal Oxide Highly Dense Nanonetworks as Free-Standing High-Capacity Anodes for Lithium-Ion Batteries
Source: ACS Appl Mater Interfaces. 2022 Jun 14;14(25):28911–23. doi: 10.1021/acsami.2c05902 (PMC9247978; doi:10.1021/acsami.2c05902)
Supplement: Supplementary file 1 — am2c05902_si_001.pdf [file am2c05902_si_001.pdf]

*Supporting information*

# 3D Monolithically Self-Grown Metal Oxide Highly-Dense Nano-Networks as Free-Standing High Capacity Anodes for Li-ion Batteries

Adam Cohen[1#], Nimrod Harpak[2#], Yonatan Juhl[1], Pini Shekhter[3], Sergei Remennik[4], and Fernando Patolsky[1,2*]

1. Department of Materials Science and Engineering, the Iby and Aladar Fleischman Faculty of Engineering, Tel Aviv University, Tel Aviv 69978, Israel.
2. School of Chemistry, Faculty of Exact Sciences, Tel Aviv University, Tel Aviv, 69978, Israel.
3. Wolfson Applied Materials Research Centre, Tel Aviv University, Tel Aviv, 69978, Israel.
4. The Center for Nanoscience & Nanotechnology, Edmond J. Safra Campus, The Hebrew University of Jerusalem, Jerusalem 91904, Israel.

# Contributed equally.

Emails: fernando@post.tau.ac.il

**Table S1** – Elemental composition of different phases before and after self-transformation of the low-grade 201 steel.

| Element | Fe (at. %) | Cr (at. %) | Mn (at. %) | O (at. %) |
|---|---|---|---|---|
| Pre-synthesis (fiber) | 71.8 | 9.62 | 18.58 | --- |
| After synthesis (fiber) | 64.76 | 12.09 | 16.61 | 6.55 |
| Nanowire network | 2.92 | --- | 37.76 | 56.96 |
| Nanowire tip | 22.15 | 1.69 | 17.51 | 58.65 |

**Table S2** – Calculation of weight percentages of the major elements in 1cm$^2$ of low-grade 201 steel. The highlighted row shows that a 3% active material loading is only a third of the total available manganese in the steel.

<table>
<tr><th>Element</th><th>Atomic fraction</th><th>Mw (g/mol)</th><th>Weight %</th><th>Mass (mg in 1 cm$^2$)</th><th>Mn:O ratio ($Mn_3O_4$)</th><th>3% active material loading (mg/cm$^2$)</th><th>Mn Content (mg/cm$^2$)</th></tr>
<tr><td>Fe</td><td>0.71</td><td>55.84</td><td>72.11131</td><td>29.060857</td><td rowspan="5">0.72</td><td rowspan="5">1.12</td><td rowspan="5">0.8064</td></tr>
<tr><td>Cr</td><td>0.17</td><td>52</td><td>16.07873</td><td>6.4797302</td></tr>
<tr><td>Ni</td><td>0.045</td><td>58.69</td><td>4.803704</td><td>1.9358927</td></tr>
<tr><td>Mn</td><td>0.065</td><td>54.94</td><td>6.495336</td><td>2.6176204</td></tr>
<tr><td>Si</td><td>0.01</td><td>28.09</td><td>0.510918</td><td>0.2059</td></tr>
</table>

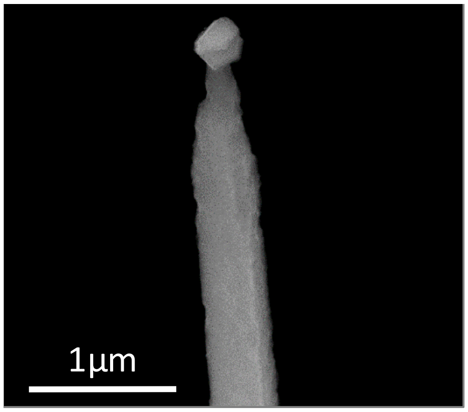


**Figure S1 -** SEM image of a single MnNW after 20 minutes growth time, exhibiting an oddly shaped particle at its tip.

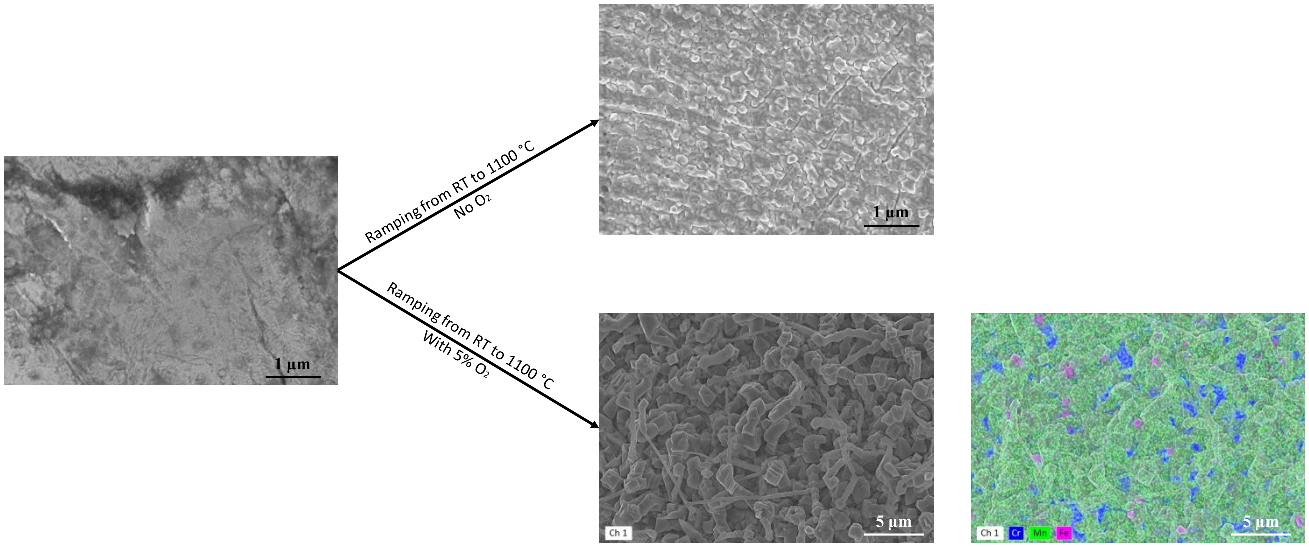


**Figure S2 –** SEM images depicting the difference in the surface of the steel after thermal ramping at different conditions.

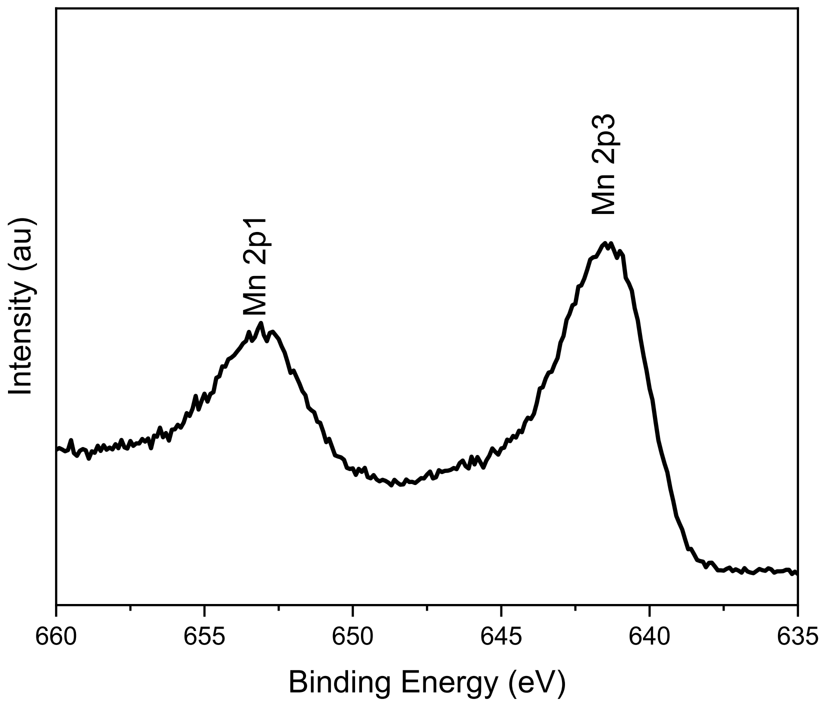


**Figure S3** – High resolution XPS spectrum depicting Mn 2p spectrum of the stainless steel after 12 hours of growth time.

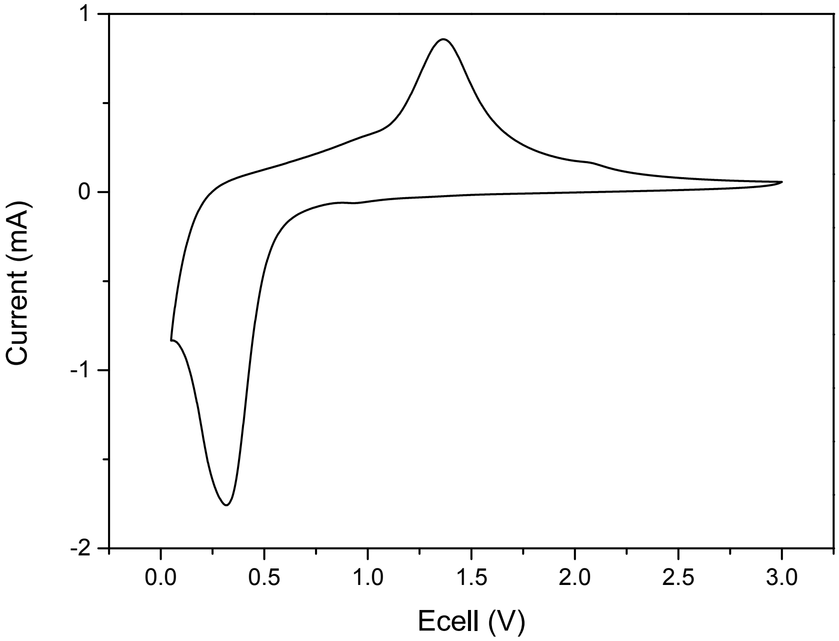


**Figure S4** – Representative cyclic voltammetry measurement of the composite $Mn_3O_4$ anode, measured at 0.2mV/s.

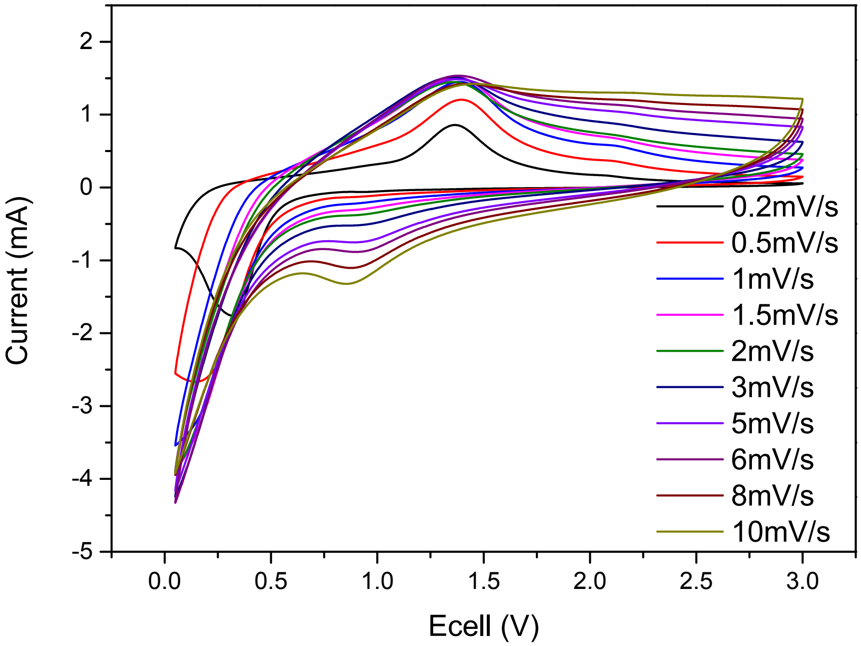


**Figure S5 –** Cyclic voltammetry measurements at different rates of the composite $Mn_3O_4$ anode.

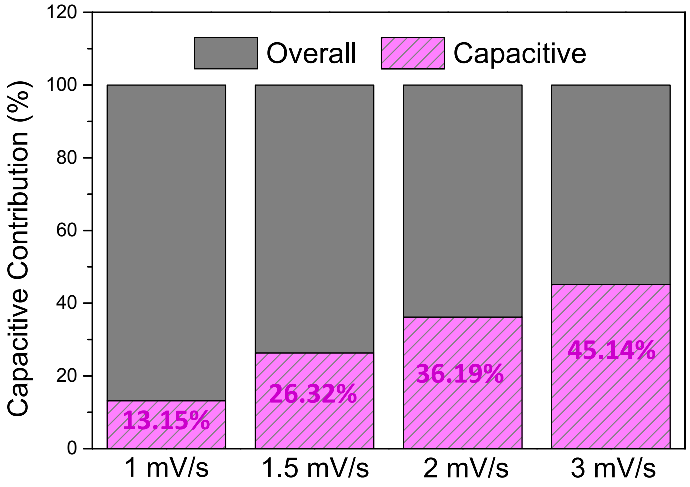


**Figure S6 –** Capacitive contributions as a function of different scan rates, calculated from CV measurements at different rates and the Dunn method.

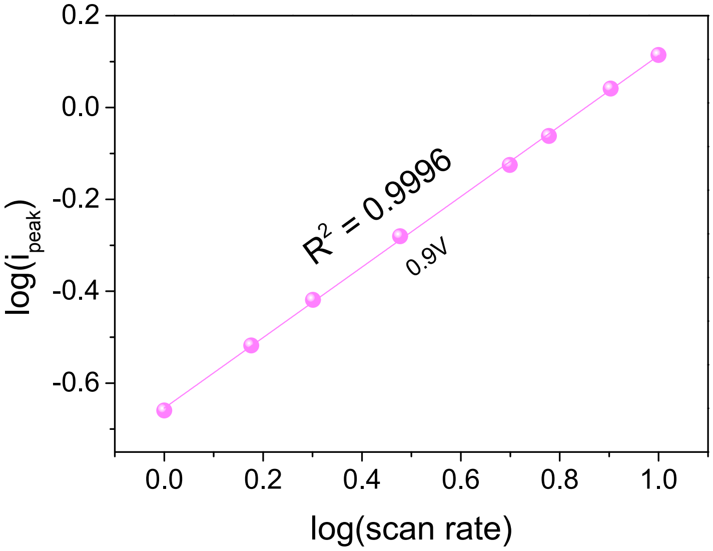


**Figure S7 –** Linear relationship of log(i) versus log(scan rate) taken from a peak found at 0.9V on the CV measurements taken at different rates.

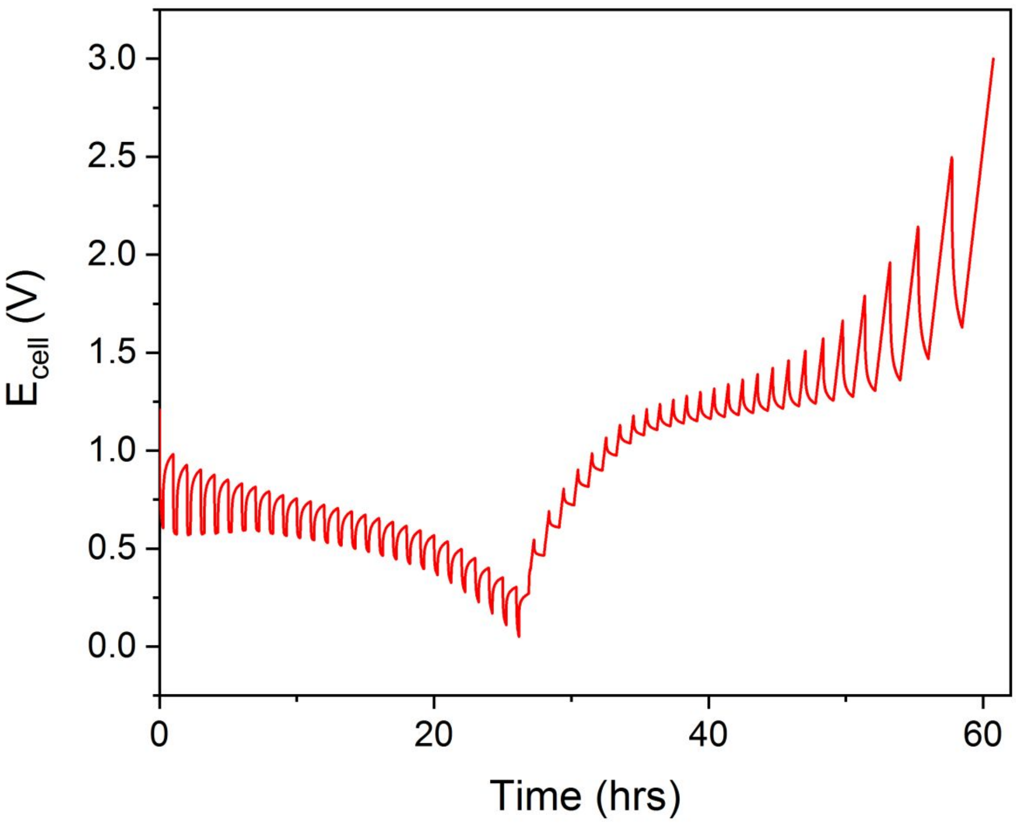


**Figure S8 –** GITT measurement performed after one complete cycle. The measurement was conducted at C/10 rate where the current was activated for 15 minutes and rest times were set to 30 minutes.
